# Aflatoxin Contamination of Milk Produced in Peri-urban Farms of Pakistan: Prevalence and Contributory Factors

**DOI:** 10.3389/fmicb.2020.00159

**Published:** 2020-03-04

**Authors:** Agha Waqar Yunus, Aman Ullah, Johanna Frida Lindahl, Zahid Anwar, Atta Ullah, Sharjeel Saif, Mubarak Ali, Aamer Bin Zahur, Hamid Irshad, Shahbaz Javaid, Nida Imtiaz, Umer Farooq, Aitzaz Ahsan, Zahida Fatima, Avais Ahmed Hashmi, Babar Hilal Ahmad Abbasi, Zubair Bari, Ihsan Ullah Khan, Mohammed Nawaz Mohammed Ibrahim

**Affiliations:** ^1^Animal Sciences Institute, PARC National Agricultural Research Centre, Islamabad, Pakistan; ^2^International Livestock Research Institute, Hanoi, Vietnam; ^3^Department of Animal Genomics and Biotechnology, PARC Institute of Advanced Studies in Agriculture, Islamabad, Pakistan; ^4^Department of Agricultural Sciences, Faculty of Sciences, Allama Iqbal Open University, Islamabad, Pakistan; ^5^Livestock and Dairy Development Department, Quetta, Pakistan; ^6^Center for Advanced Studies in Vaccinology and Biotechnology, University of Balochistan, Quetta, Pakistan; ^7^Livestock and Dairy Development Department, Lahore, Pakistan; ^8^Livestock and Dairy Development Department, Peshawar, Pakistan; ^9^International Livestock Research Institute (ILRI), Islamabad, Pakistan

**Keywords:** aflatoxin, cottonseed, dairy, feed, milk

## Abstract

Aflatoxin M_1_ contamination of milk in Pakistan, like many developing countries, is poorly understood. The present study was therefore conducted to determine AFM_1_ contamination of milk and its contributory factors in Pakistan. We sampled milk and feedstuffs from 450 peri-urban dairy farms in seven major cities following a cross-sectional study design. Analysis of milk using ELISA revealed high contamination with an overall average of 3164.5 ng of AFM_1_/L, and significant differences (*p* < 0.001) between cities. The milk sampled from Gilgit, in northern hilly areas, had an average AFM_1_ level of 92.5 ng/L. Milk from other cities had 3529.7 ng/L average contamination, with only 5.7% samples qualifying the maximum tolerable limit of 500 ng of AFM_1_/L. Heavy mean aflatoxin contamination was found in bakery waste (724.6 μg/kg), and cottonseed cake (600.8 μg/kg). Rest of the other feedstuffs had moderate to low mean aflatoxin contamination, ranging from 66.0 μg/kg in maize stover to 3.4 μg/kg in wheat bran. The mean aflatoxin level in commercial dairy concentrates was 32.7 µg/kg. About 80% of the total aflatoxin intake of dairy animals was contributed by cottonseed cake alone due to its high aflatoxin contamination and proportion in dairy rations. On-farm storage time of oilseed cakes varied (*p* < 0.01) in different cities but was not associated with aflatoxin contamination. The exceptionally high AFM_1_ contamination suggests that milk from peri-urban dairy farms is a serious public health threat in Pakistan. This situation can be mitigated by reducing aflatoxin contamination in cottonseed cake and promoting the use of commercial concentrates and other feedstuffs with low contamination.

## Introduction

Aflatoxins are toxic secondary metabolites of various *Aspergillus* spp. that commonly contaminate agricultural produce worldwide. The four main forms of aflatoxins encountered in grains and other commodities are B_1_, B_2_, G_1_, and G_2_. Once ingested by animals, the aflatoxin B_1_ and G_1_ are excreted in the form of aflatoxin M_1_ (AFM_1_) in milk and eggs. Majority of the aflatoxins in food and feed occur in the form of B_1_ and therefore AFM_1_ is the mainly encountered form in animal products (Yunus et al., 2011). All these forms of aflatoxins are hepatotoxic and carcinogenic in nature. Therefore, their levels in food and feed are regulated in over 100 countries (Food and Agriculture Organization, 2004). The toxicity of aflatoxins is higher in younger age groups, and stunted growth due to aflatoxin contamination of foods has been suggested (Khlangwiset et al., 2011). Monitoring of aflatoxins in baby foods and milk is therefore more critical.

Pakistan has a climate that typically favors development of aflatoxins in foods. Several authors have reported aflatoxin contamination of various foods in the country during the last two decades (review, Ashiq, 2015). The government of Pakistan however only recently introduced legislation on aflatoxin levels in foods and feeds. Pakistan Quality and Standards Control Authority now allows 500 ng/L as the maximum allowed limit of AFM_1_ in milk (amendment 2 in standard PS-5344-2016). In practice, this restriction is only followed by milk processors which have merely 5% share in the total milk marketed in the country.

Research on AFM_1_ contamination of milk in Pakistan shows high variations in contamination levels. In this regard, Maqbool et al. (2009) and Iqbal et al. (2011) found mean AFM_1_ levels to be 41 and 46 ng/L, respectively in milk sampled from major cities in two provinces. Similarly, Hussain (2009) found the average AFM_1_ levels to vary from 199 to 503 ng/L in different seasons in milk sampled from 14 districts of Punjab province. In the latter study, only 3% samples were found to exceed the 500 ng of AFM_1_/L limit, while the former two authors did not find any sample to exceed this limit. Contrary to these studies, Aslam et al. (2016) found 87% of milk samples to exceed the 500 ng/L limit in three districts of Punjab province. Likewise, Akbar et al. (2019) found 69% milk samples from different regions of the Punjab province to exceed the allowed limit. In one study, mean AFM_1_ contamination in Lahore city was found to be as high as 17,380 ng/L with 81% samples exceeding the 500 ng/L limit. Similar to the case of Pakistan, high variability in aflatoxin levels has been noted in some other countries. In Kenya, a country where aflatoxins have been extensively studied, variation in AFM_1_ contamination has been reported depending upon agroecological zones, seasons, and even type of milk product (Senerwa et al., 2016; Lindahl et al., 2018). In case of Pakistan, the studies conducted to date differ not only in the sampling area and season, but also in the methods employed to quantify AFM_1_. Some of these studies were conducted on a very limited scale, which limits the application of their results to the overall situation in the country. In this scenario, we recently investigated seasonal variation in levels of AFM_1_ in processed and raw milk in Pakistan (Yunus et al., 2019). The results indicated that raw milk is routinely contaminated with high levels of AFM_1_ and that the levels are in general higher during winter months. The present study was therefore conducted as the first nationwide investigation on AFM_1_ contamination of raw milk in peri-urban dairy farms of Pakistan during the high season (winter months). The aim was to not only identify areawise AFM_1_ contamination of milk, but to also identify the factors that contribute to milk contamination.

## Materials and Methods

### Sampling

**Figure 1:**
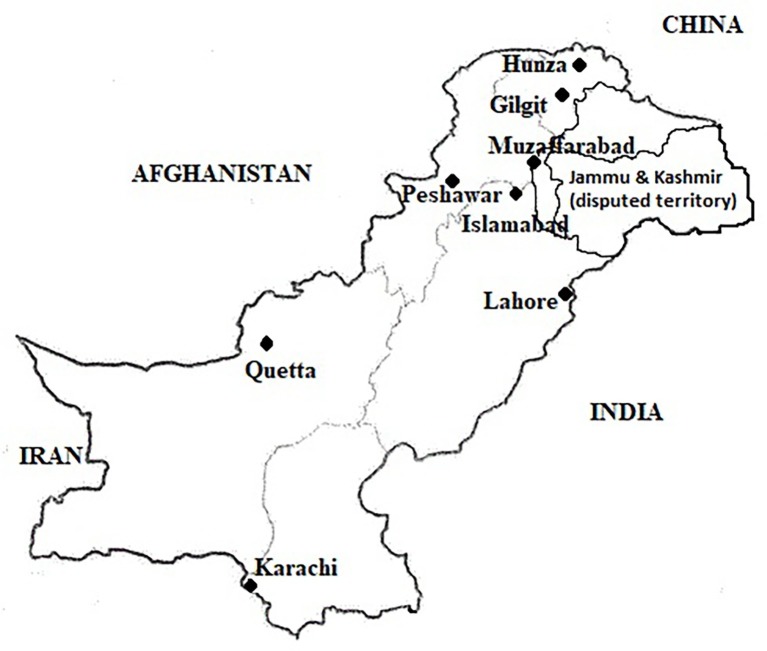
Geographical distribution of sampling sites within Pakistan.

A cross-sectional survey was conducted and pooled milk samples were collected from peri-urban dairy farms in all provincial/regional capitals in Pakistan from October to mid-December 2016. Assuming a simple random sampling, the calculated sample size was 384 milk samples with 50% proposed prevalence, 95% confidence interval, and 80% power/precision of the study (http://www.winepi.net). However, sample size was increased by approximately 10% to accommodate for any losses of milk samples during storage/transportations. Further, sample size for each city was calculated using stratified random sampling using probability proportional to size (PPS). A peri-urban dairy farm was defined as a farm located within the boundaries of identified city districts with a minimum herd size of 2 milch animals intended for sale of milk. This inclusion criterion was followed for all the cities except Gilgit, where it was relaxed to one milking animal due to small herd sizes in the city. The livestock population data (including herd size in different cities) and expert opinion of the Provincial Livestock and Dairy Development departments were considered about the number of peri-urban dairy farms in the city for sample size calculations. Finally, we planned sampling from 450 farms all across Pakistan (Islamabad = 75, Karachi = 70, Lahore = 90, Quetta 50, Peshawar = 75, Muzaffarabad = 50, and Gilgit = 40) (Figure 1). In case of the 40 samples from Gilgit, 13 samples were collected from Hunza valley, which is situated further toward north. Although information was collected from all the farms, only 372 raw milk samples could be tested.

Information on the relevant husbandry practices regarding feeding, and procurement and storage of feed was recorded from the farmers. From each farm, 500 ml of milk was sampled from the bulk milk tank. From this 500 ml sample, an aliquot of 50 ml and two further aliquots of 15 ml were separately taken in falcon tubes. Milk samples were kept refrigerated, and without any addition of preservatives, until reaching the lab where these were frozen at −20°C until analysis. On the day of milk sampling, samples of all the dry feedstuffs being used at the farms were also collected. These included wheat straw, maize stover, oilseed cakes (cotton, canola, palm, coconut, and maize), legumes (various pulses), grains (maize, and wheat), brans (wheat bran, and pulse bran), rice polish, corn gluten, dates, commercial concentrates, waste bread, and bakery waste. For each feedstuff, 6–10 incremental samples of 100 g each were collected using sampling probes. The aggregate sample thus obtained was reduced to 250 g laboratory sample after thorough mixing, and carried in paper bags to the laboratory.

During the same time, all the brands of UHT milk (*n* = 15), pasteurized milk (*n* = 13), local milk powder (*n* = 4), and imported milk powder (*n* = 13) were also sampled from Islamabad. Except for the imported milk powder, the results regarding processed milk have already been published (Yunus et al., 2019). In the present report, these results are being presented for comparison purposes.

### Aflatoxin Analyses

Milk samples were analyzed for AFM_1_ contamination using ELISA kits (AFM-E01, Immunolab GmbH, Kassel, Germany) following protocols specified by the manufacturer. The kit had a quantification range of 10–1,000 ng of AFM_1_/L. Milk samples were analyzed several times in different dilutions until the AFM_1_ levels in the diluted samples fell within the quantification range of the ELISA kit, as detailed earlier for a sister study (Yunus et al., 2019). The samples were first analyzed at either 1X or 2X dilution, and then depending upon the optical density (OD) values, these were analyzed again after dilution at 3, 4, 5, 6, 8, 10, 12 15, or 20X. Analysis of some samples had to be repeated up to five times in different dilutions to get OD values within the range of the ELISA kit.

The feed samples were analyzed for total aflatoxin levels using ELISA kits (AFT-E01, Immunolab, Kassel, Germany) following protocols specified by the manufacturer. The kit for total aflatoxin analysis had a quantification range of 1.75–52.5 μg of aflatoxins/kg sample. Samples were extracted in 70% methanol and appropriately diluted to fall within the quantification range of the kit.

Kits were read on an ELISA reader (BDSL, Immunoskan MS 355, Labsystems, Vantaa, Finland). Aflatoxins in milk and feedstuffs were quantified using a software based on four parametric curve estimations provided by the manufacturer of the ELISA kits.

### Quality Control in Aflatoxin Analyses

The ELISA kit for AFM_1_ analysis was validated before start of this study (Imtiaz and Yunus, 2019). In addition, 4.4, and 44 ng of AFM_1_/L external standards made using a reference skim milk powder (RMBD-248, EU Joint Res Center, IRMM, Geel, Belgium), and 50, and 500 ng of AFM_1_/L external standards made using a purified 9.786 μg of AFM_1_/ml solution (46,319 U, Supelco, Bellefonte, PA, USA) were run on each microtiter plate as described earlier (Yunus et al., 2019). For quality control during total aflatoxin analysis in feedstuffs, external standards were prepared diluting a certified standard having 3.228 μg of AFB_1_/ml (catalogue numb 46,323 U, Supelco, USA).

Recovery of AFM_1_ was 86.9% at 500 ng of AFM_1_/L, while recovery of total aflatoxins at a reference value of 22.4 μg/kg of cornmeal was 109.4%. Results were not corrected for recovery.

### Data Analyses

The percent contribution of feedstuffs to total aflatoxin exposure of animals in each city was calculated using following equation:

Percent contribution of a feedstuff in a city = (Average aflatoxin intake from a specific feedstuff per animal ÷ average of total aflatoxin intake from all feedstuffs per animal) × 100, where the aflatoxin intake from a specific feedstuff per animal was calculated as a multiple of average daily consumption of the feedstuff per animal and its average aflatoxin contamination for each city.

The data are presented as arithmetic means, and were statistically analyzed by applying ANOVA and least significant difference test. Differences were considered significant at *p* < 0.050. The Pearson correlation coefficients (r) between AFM_1_ level in milk and use of various feed ingredients were determined. All statistical analyses were conducted using IBM SPSS Statistics 20 (IBM Corp., Armonk, New York, NY, USA, 2011).

## Results and Discussion

### Aflatoxin M_1_ Levels in Milk

The AFM_1_ levels in milk collected from peri-urban dairy farms in different cities are presented in Table 1. Differences between AFM_1_ contaminations in different cities were significant (*p* < 0.001). The milk sampled from Islamabad, Lahore, and Muzaffarabad was found to have a mean concentration of 4,799.6 ± 3,945.5 ng of AFM_1_/L. The levels of AFM_1_ in these cities were higher (*p* = 0.006) than the levels of the toxin in milk sampled from other cities. Milk sampled from Karachi, Peshawar, and Quetta was found to have an average of 1946.8 ± 1562.9 ng of AFM_1_/L. These three cities had higher (*p* = 0.019) AFM_1_ contamination than the milk sampled from Gilgit.

**Table 1:** Aflatoxin contamination of milk produced in various cities.

City	*n*	AFM_1_ level (ng/L)				>50 ng/L	>500 ng/L	>1,000 ng/L
		Mean ± SD	Min	Max	Median			
Islamabad	69	4935.3 ± 3468.7^a^	417.7	15636.1	4123.1	100%	97.1%	89.9%
Lahore	83	4842.6 ± 4310.5^a^	311.8	15994.2	3194.6	100%	96.4%	83.1%
Muzaffarabad	35	4436.1 ± 4068.2^a^	554.3	13525.7	2990.2	100%	100%	82.9%
Karachi	32	2435.9 ± 1740.3^b^	772.3	7966.4	1853.1	100%	100%	81.2%
Peshawar	69	1930.8 ± 1626.9^b^	138.9	8789.5	1376.0	100%	85.5%	60.9%
Quetta	45	1623.5 ± 1242.5^b^	254.2	4997.1	1247.8	100%	91.1%	62.2%
Gilgit	39	92.5 ± 178.6^c^	<LOD	796.1	26.1	35.9%	7.7%	0.0%
Overall	372	3164.5 ± 3405.3	<LOD	15994.2	1904.6	93.3%	85.2%	68.8%

*Means bearing different superscripts “^abc^” differ (p ≤ 0.01) within the column. LOD < 4.4 ng AFM_1_/L; LOQ = 10 ng AFM_1_/L*.

While only 3% samples in Islamabad and Lahore qualified the 500 ng of AFM_1_/L limit, no sample in Muzaffarabad could qualify this limit. Also, over 80% sample in these cities were higher than 1,000 ng of AFM_1_/L. Despite a lower level of contamination in Karachi, none of the samples in this city qualified the 500 ng/L limit, and over 80% samples had levels higher than 1,000 ng/L. Compared to these cities, 10–15% samples in Peshawar and Quetta had AFM_1_ levels lower than 500 ng/L, and 40% samples had lower than 1,000 ng/L. The average level of AFM_1_ in milk sampled from Himalayan city of Gilgit was 92.5 ± 178.6 ng/L and 46% samples here were even lower than the limit of 50 ng/L followed by the EU. Only 7.7% samples in Gilgit exceeded the 500 ng/L limit. In this regard, it would be worth mentioning that as the sampling area went further north in Hunza valley, the mean AFM_1_ content decreased to only 10.9 ng/L and all the samples here were below the 50 ng of AFM_1_/L limit followed by the EU.

During the same time (Oct–Nov), all the major brands of processed milk were collected for comparison. Results presented in Table 2, and as also partially reported earlier by us (Yunus et al., 2019), indicated that 54% of the milk powder brands based on imported milk powder were below the 50 ng of AFM_1_/L limit, while the remaining others qualified the 500 ng/L limit. On the contrary, none of the local brands qualified the 50 ng/L limit. Only one out of four samples in this latter group qualified the 500 ng/L limit. These results are alarming as the end users of these milk powders are infants, who are more sensitive to AFM_1_ contamination compared to the older age groups. It is interesting to note that UHT milk during the same time had a mean AFM_1_ contamination level of 366 ng/L with 73% samples qualifying the 500 ng/L AFM_1_ limit. Ironically, the UHT brands of the companies, whose baby milk powder brand exceeded the accepted standards, qualified the 500 ng AFM_1_/L limit. These results indicate that either there is lack of knowledge or the raw milk with higher AFM_1_ contamination is being channeled to production of milk powder due to lesser quality control on such products. The pasteurized milk collected during this time exceeded the accepted standards and had higher (*p* ≤ 0.007) AFM_1_ levels than UHT and imported milk powder. On an overall basis, the liquid processed milk (UHT and pasteurized combined) had an average AFM_1_ contamination of 738.0 ng/L, which was found to be lower (*p* < 0.001) than the contamination in raw milk from Islamabad, Lahore, and Muzaffarabad. The liquid processed milk was also found to have lower (*p* = 0.023) AFM_1_ contamination than Karachi. Statistically, the AFM_1_ contamination in processed milk was not found to be different (*p* ≥ 0.064) than the contamination in milk sampled from Quetta, Peshawar, and Gilgit.

**Table 2:** Aflatoxin contamination of processed milk during October to November.

Type of processed milk	*n*	AFM_1_ level (ng/L)^1^				>50 ng/L	>500 ng/L	>1,000 ng/L
		Mean ± SD	Min	Max	Median			
Imported milk powder^1^	13	58.4 ± 31.5^b^	7.3	121.9	56.5	46.1%	0.0%	0.0%
Local milk powder^1^^,^^2^	4	922.5 ± 690.5^abc^	412.5	1935.0	671.4	100.0%	75.0%	25.0%
UHT^2^	15	365.7 ± 168.0^b^	145.5	642.9	346.5	73.3%	26.7%	0.0%
Pasteurized^2^	13	1167.5 ± 1333.7^a^	56.9	3935.5	454.5	100.0%	36.4%	45.5%
Overall	45	558.1 ± 857.3	7.3	3935.5	395.8	66.7%	24.4%	13.3%

*Means bearing different superscripts “^abc^” differ (p ≤ 0.01) within the column*.

1 Milk powder reconstituted at 15 g per 115 ml for AFM_1_ analysis;

2 Monthwise averages for local milk powder, UHT, and pasteurized milk have been reported previously (Yunus et al., 2019).

The presently reported AFM_1_ levels in milk are very high compared with some of the previous reports (Raza, 2006; Iqbal et al., 2011, 2014; Younus et al., 2013; Ahmad et al., 2019), but lower than the levels reported by Muhammad et al. (2010) for Lahore city. The contamination levels are also higher compared to what has been found in milk in different parts of Africa (Ayalew et al., 2016). It should be noted in this regard that the samples in the present study were collected in a season that is associated with higher contamination of milk. Secondly, samples were collected from peri-urban dairy farms, which use lesser fodder compared with the farmers in villages. Such practices are associated with higher levels of AFM_1_ in milk (Iqbal et al., 2014). In addition, some of the previous authors from Pakistan such as Ahmad et al. (2019) and Maqbool et al. (2009) relied on methods that only allow AFM_1_ quantification up to 100 ng/L. Similarly, Younus et al. (2013) who reported median AFM_1_ concentration of 333 and 416 ng/L in summer and winter months, respectively, in district Jhang used a method (snap AFM1, by IDEXX) that only allows quantification up to 500 ng/L. Such methods may not be appropriate for accurate quantification of AFM_1_ contamination.

### Usage of Dairy Feedstuffs

Data regarding share of different feedstuffs in total dry matter (DM) fed to the dairy animals in different cities are presented in Tables 3, 4. All feed ingredients were used at different proportions (*p* ≤ 0.018) in different cities. The share of cottonseed cake in total DM correlated positively (*r* = 0.31; *p* < 0.001) with the AFM_1_ levels in milk in different cities. Number of farms using cottonseed cake were less in the cities with low AFM_1_ contamination (Giglit). Statistically, there was a positive association (*r* = 0.36; *p* < 0.001) between incidence of cottonseed cake use and the AFM_1_ levels in milk.

**Table 3:** Percentage (%) of various protein sources in total DM in different cities.

City	Oilseed cakes					Legumes				Mixed concentrate	
	Cotton cake	Brassica cakes	Maize cake	Palm cake	Coconut cake	Pulses	Cowpea	Mung beans	Waste pulses	Comr.	Home mix
Islamabad	16.7^a^	1.9^a^	0.0^b^	0.0^b^	0.0^b^	0.0^b^	0.0^b^	0.0^b^	0.0^b^	2.2^c^	0.0
Lahore	8.1^b^	0.2^c^	0.0^b^	0.0^b^	0.0^b^	0.0^b^	0.0^b^	0.0^b^	0.0^b^	1.5^c^	0.0
Muzaffar.	18.5^a^	0.0^c^	0.0^b^	0.0^b^	0.0^b^	0.0^b^	0.0^b^	0.0^b^	0.0^b^	8.3^a^	0.0
Karachi	1.3^c^	0.6^bc^	0.0^b^	7.7^a^	0.8^a^	1.2^a^	0.0^b^	0.3^a^	3.6^a^	6.7^a^	3.9^a^
Peshawar	8.1^b^	0.3^c^	2.3^a^	0.0^b^	0.0^b^	0.0^b^	0.0^b^	0.0^b^	0.0^b^	6.1^ab^	0.0
Quetta	9.4^b^	2.9^a^	0.0^b^	0.2^b^	0.0^b^	0.5^b^	1.5^a^	0.0^b^	0.4^b^	0.6^c^	0.0
Gilgit	0.9^c^	1.8^ab^	0.0^b^	0.0^b^	0.0^b^	0.0^b^	0.0^b^	0.0^b^	0.0^b^	3.3^bc^	0.0
Total:	9.27	1.00	0.41	1.17	0.12	0.24	0.18	0.49	0.60	4.03	0.59
SD	9.39	3.32	1.86	3.42	1.02	1.49	1.50	0.53	2.38	8.57	3.89
*p*	0.001	0.001	0.001	0.001	0.001	0.001	0.001	0.001	0.001	0.001	0.001
*n*	429	429	429	429	429	429	429	429	429	429	429

*Means with different superscripts “^abc^” differ significantly within a column at p ≤ 0.05. Muzaffar. = Muzaffarabad; Brassica cakes = mustard oilseed cake, taramira (Eruca sativa) seed cake, or canola meal; Pulses = any out of chick pea, or other Indian pulses; cowpea = local variety of cow pea (matar dana); mong beans = mong kata; waste pulses = mix ati; home mix = pala; Comr. = commercial*.

**Table 4:** Percentage (%) share of various energy sources, brans, and roughages in total dry matter.

City	Energy sources						Polish and brans			Roughages^*^			Misc. stuffs
	Wheat	Maize	Waste bread	Bakery waste	Dates	Oil	Rice polish	Wheat bran	Pulses bran	Straw	Fodd.	Stov.	
Islamabad	1.8^cd^	0.1^c^	15.0^a^	0.0^c^	0.1^b^	0.03^b^	0.0^c^	8.0^de^	2.1^a^	36.4^b^	15.7^c^	0.1^b^	0.0^c^
Lahore	2.7^c^	0.1^c^	9.5^b^	0.0^c^	0.0^b^	0.0^b^	0.0^c^	6.0^e^	0.1^b^	17.1^d^	47.3^a^	2.8^a^	1.5^c^
Muzaffar.	0.4^d^	0.4^bc^	2.4^c^	0.0^c^	0.0^b^	0.0^b^	0.0^c^	13.6^bc^	0.0^b^	44.5^a^	0.0^d^	2.1^ab^	9.8^b^
Karachi	7.0^b^	0.9^b^	3.4^c^	1.1^a^	0.3^a^	0.13^a^	1.6^b^	14.0^b^	0.3^b^	31.7^b^	12.7^c^	0.0^b^	0.1^c^
Peshawar	0.5^d^	0.1^c^	3.4^c^	0.6^b^	0.0^b^	0.001^b^	0.0^c^	11.0^bc^	0.0^b^	36.1^b^	30.9^b^	0.0^b^	0.5^c^
Quetta	2.9^c^	2.6^a^	12.7^a^	0.3^bc^	0.3^a^	0.01^b^	8.2^a^	10.4^cd^	0.0^b^	31.7^b^	10.7^c^	0.0^b^	2.5^c^
Gilgit	14.3^a^	0.0^c^	2.8^c^	0.0^bc^	0.0^b^	0.0^b^	0.0^c^	19.4^a^	0.0^b^	23.5^c^	0.0^d^	3.0^a^	30.9^a^
Total	3.63	0.55	7.45	0.30	0.09	0.03	1.22	11.02	0.42	31.37	19.76	1.03	4.61
SD	6.99	2.37	9.48	1.57	0.70	0.16	3.57	10.03	2.17	16.49	21.43	6.88	14.88
*p*	0.001	0.001	0.001	0.001	0.009	0.001	0.001	0.001	0.001	0.001	0.001	0.018	0.001
*n*	429	429	429	429	429	429	429	429	429	429	429	429	429

*Means with different superscripts “^abcd^” differ significantly within a column at p ≤ 0.05. Muzaffar. = Muzaffarabad; Fodd. = Fodder; Stov. = stover; straw = wheat straw; Pulse bran = sorhi*.

* *Share of grazing from Lahore not included*.

The amount of cottonseed cake fed per animal was also positively (*r* = 0.31; *p* < 0.001) associated with AFM_1_ contamination of milk (data not shown here). The highest AFM_1_ levels were found in milk from Islamabad, Lahore, and Muzaffarabad, where cottonseed cake was the sole oilseed cake used. As cottonseed cake was replaced with other oilseed cakes in other cities, the AFM_1_ levels decreased in milk. In this regard, the amount of commercial concentrates fed per animal was negatively correlated (*r* = −0.12; *p* = 0.026) with AFM_1_ level of milk. The combined amount fed per animal of Brassica meals, maize oilcake, palm oilcake, and coconut oilcake was also found to have low and negative correlation (*r* = −0.14; *p* = 0.008) with the AFM_1_ level of milk.

In case of energy sources, the amount of waste bread fed per animal was found to have positive but low correlation (*r* = 0.16; *p* = 0.002), while amount of wheat and maize grains was found to have negative correlation (*r* = −0.20; *p* < 0.001) with AFM_1_ contamination. The share of grains (wheat and maize) in total DM also showed a low but negative (*r* = −0.27; *p* < 0.001) correlation with the AFM_1_ levels in milk. Besides grains, the share of wheat bran in total DM also had a low negative correlation (*r* = −0.16; *p* = 0.003) with AFM_1_ contamination. Its share was lowest, i.e., 8.0 and 6.0% of DM in the two cities with highest AFM_1_ levels while highest, i.e., 19.4% of DM in Gilgit, which had lowest AFM_1_ contamination. In case of brans and polish, the share of pulse bran in total DM fed to animals was found to have positive correlation (*r* = 0.10; *p* = 0.049), while rice polish was found to have negative correlation (*r* = −0.14; *p* = 0.007) with AFM_1_ in milk.

Overall, significant but low correlations of some feed ingredients were found with AFM_1_ contamination of milk, indicating that each ingredient contributed to AFM_1_ levels according to its percentage in the total DM fed to the animal. Another interesting feature found from these data was that the more the number of ingredients used in dairy ration formulation, the less was the AFM_1_ contamination (except for Gilgit).

### Aflatoxin Contamination of Dairy Feedstuffs

Data regarding the aflatoxin contamination of dairy feedstuffs in different cities are presented in Tables 5, 6. Bakery waste was the most contaminated feedstuff with an average level of 724.6 μg of aflatoxins/kg. However, only 17 farmers (3.8% of total 448) in Karachi, Quetta, and Peshawar were using bakery waste and that too at an average 0.6 to 1.1% of the total ration’s DM. The second highest aflatoxin contamination, 595.9 μg/kg, was recorded in cottonseed cake, which was being used by 64.7% of the farmers and at an average rate of 9.3% of the total DM. In Islamabad, Lahore, Muzaffarabad, Peshawar, and Quetta, the average use of cottonseed cake was 17.0, 8.4, 18.5, 7.9, and 9.2% of the total DM, respectively. In these cities, use of cottonseed cake could explain the variation in AFM_1_ levels in milk. The share of cottonseed cake in total ration DM in Lahore was only around half of that in Islamabad (Table 3) but its contamination level was around double of that in Islamabad. These figures offer reasonable explanation of the comparable milk contamination in Lahore and Islamabad.

**Table 5:** Total aflatoxin level (μg/kg) in different protein sources in different cities.

City	Oilseed cakes					Legumes				Mix concentrate	
	Cotton cake	Brassica cakes	Maize cake	Palm cake	Coconut cake	Pulses	Cowpea	Mung beans	Waste pulses	Commr.	Home mix
Islamabad	599.7^b^	10.7^b^	—	—	—	—	—	—	—	37.9	—
Lahore	1174.9^a^	777.9^a^	—	—	—	—	—	—	—	23.9	—
Muzaff.	397.3^b^	—	—	—	—	—	—	—	—	30.9	—
Karachi	183.0^b^	5.7^b^	—	8.0	4.0	19.3	—	15.1	40.5	28.3	15.6
Peshawar	488.8^b^	ND	45.9	—	—	—	—	—	—	33.9	—
Quetta	424.7^b^	7.1^b^	—	5.0	—	5.7	4.7	—	—	38.4	—
Gilgit^*^	31.0	8.3^b^	—	—	—	—	—	—	—	28.3	—
Total	600.84	54.87	45.87	7.68	3.97	15.94	4.75	15.09	40.55	32.68	15.59
STD	627.00	263.33	45.45	10.37	4.22	14.50	1.03	18.27	31.99	25.45	5.97
*p*	0.001	0.001	—	0.710	—	0.531	—	—	—	0.980	—
*n*	189	39	14	21	5	4	2	2	8	52	2

*Means with different superscripts “^ab^” differ significantly within a column at p ≤ 0.01. ND = not determined due to unavailability of sample; Muzaff = Muzaffarabad; Brassica cakes = mustard oilseed cake, taramira (Eruca sativa) seed cake, or canola meal; Pulses = any out of chick pea, or other Indian pulses; cowpea = local cowpea variety; commr. = commercial*.

* *Only one farmer in Gilgit used cottonseed cake, and therefore Gilgit was not included in post hoc test*.

**Table 6:** Total aflatoxin level (μg/kg) in different energy sources and roughages.

City	Energy sources				Polish and brans			Roughages		Misc. feedstuffs
	Wheat	Maize	Waste bread	Bakery waste	Rice polish	Wheat bran	Pulses bran	Straw	Stover	
Islamabad	7.4	8.9	46.4	—	—	1.9^b^	49.1	4.4	ND	—
Lahore	34.7	ND	15.5	—	—	2.6^b^	ND	6.0	156.7	351.0^a^
Muzaffar.	—	ND	192.1	—	—	2.3^b^	—	5.0	25.5	32.5
Karachi	21.0	61.6	28.5	31.8	5.3	ND	4.7	ND	—	<LOD
Peshawar	51.5	ND	155.2	1556.1	—	1.8^b^	—	4.6	—	—
Quetta	16.0	27.7	4.6	—	25.4	3.8^b^	—	12.0	—	<LOD
Gilgit	6.4	—	8.0	—	—	6.2^a^	—	—	2.3	5.7^b^
Total	17.48	28.93	61.61	724.63	22.01	3.37	45.36	6.29	66.00	67.93
SD	33.31	49.32	219.74	1957.96	31.56	3.21	34.30	10.74	135.97	184.46
*p*	0.127	0.773	0.079	0.215	0.101	0.001	0.232	0.595	0.396	0.001
n	57	12	124	11	47	87	12	42	8	29

*Means with different superscripts “^ab^” differ significantly within a column at p ≤ 0.01. Aflatoxin not analyzed in dates, oil, and fodder. Muzaffar. = Muzaffarabad; Pulses bran = sorhi; ND = not determined*.

Cottonseed cake in Lahore had higher (*p* ≤ 0.005) aflatoxin contamination compared to other cities. The other oilseed being used in Lahore was canola meal and it was also found to have higher (*p* ≤ 0.001) levels of aflatoxins than in other cities. This trend suggests that the environmental and management conditions in Lahore may not be suitable for storage of oilseed cakes, at least during the study year. Same was true for maize stovers and crushed wheat samples from Lahore as both of these ingredients had higher levels of aflatoxins than in other cities. The other ingredients collected from Lahore such as wheat bran, wheat straw, and waste bread were found to have aflatoxin levels comparable to other cities. Presently reported results regarding aflatoxin contamination of cottonseed cake are in line with our previous report (Yunus et al., 2015). In that study, 556–5,574 μg of total aflatoxins/kg was reported using LC–MS/MS methods. Similarly, Ullah et al. (2016) reported mean aflatoxin B_1_ levels in cottonseed cake as 137.1 μg/kg using IAC-HPLC-Flu method. As found by these authors, the levels in cottonseed cake were 11 times higher than the levels in commercial feed for goats, while 24 times higher than the levels in wheat bran.

Present results on aflatoxins in cottonseed cake are however not in line with the study of Hussain (2009) who reported AFB_1_ levels in dairy feeds in various cities of Punjab. Using HPLC-Flu methods, he found the AFB_1_ levels in cottonseed cake to vary between 11 and 861 μg/kg with a mean of 242 μg/kg. The author reported AFB_1_ levels in dairy concentrate, wheat bran, and waste bread to be 176.3, 98.4, and 23.4 μg/kg, respectively. In the present study, the levels of aflatoxins in cottonseed cake are higher than found in the study of Hussain (2009) but the overall trend is similar. The levels of aflatoxins in concentrate mixes were lower in the present study, which could be an indication of improved quality control by feed manufacturers in the recent years.

Among all the tested ingredients, wheat bran was found to be the safest regarding aflatoxin contamination. Wheat bran in Gilgit was found to have 8.6 μg/kg average aflatoxin level, which was higher (*p* ≤ 0.027) than other cities. However, these levels are still within the acceptable limit of 20 μg/kg for any dairy feed ingredient. These results are not consistent with the earlier reports in which wheat bran was reported to have higher levels of mycotoxins than wheat grains (Bandara et al., 1991; Schatzmayr and Streit, 2013). It may be possible that the source of wheat bran in the present study was flour mills that produce it for human consumption and therefore might use better quality grains. The wheat grains used by farmers for dairy animal feeding are on the other hand usually from the produce that is not bought by the government due to quality issues.

The other safer feedstuffs were legumes/pulses, i.e., mung beans, cow peas (*matar dana*), and miscellaneous pulses (including chick pea etc). Contrary to these, the waste pulses (*mix ati*) used specifically for dairy animals in Karachi were found to have high levels of aflatoxins. The reason could once again be the quality and intended use of the feedstuff. While, the mung beans and other miscellaneous pulses are basically intended for human use, the pulse waste comprises of the leftover portions of various legumes/pulses and is rated not-fit for human consumption. This includes damaged legume grains and fiber portions of the legumes, besides frequent contamination with soil.

It is a general opinion of the farming community and the field veterinary officers that maize oilcake is safe for dairy animal feeding, and has very low toxin content. In the present study, maize oilcake was found to have lower levels of aflatoxins compared to cottonseed cake, but these levels were still not safe for dairy animal feeding, i.e., almost double than the allowed limit of 20 μg/kg. The palm and coconut oilcakes were however found to be good choices for dairy feeding. These are imported from Malaysia and are only used in Karachi for dairy feeding. It is therefore recommended that these two ingredients are introduced in other cities as partial replacement of cottonseed cake. Other oilseed meals/cakes like soybean can be included in rations to improve the quality.

Crushed maize grains were found to be used only by the farmers in Karachi, Islamabad, and Quetta. Except for Karachi and few contaminated samples in Quetta, maize grains were found to be within the safe limits. Not much samples could be collected from Karachi and therefore the data regarding Karachi may not represent the true picture in the city.

### On-Farm Storage of Feedstuffs

The data regarding on-farm storage of various feedstuffs in different cities are presented in Table 7. Significant differences in on-farm storage times were noted for oilseed cakes, mixed concentrates, waste bread, wheat bran, and wheat straw. In general, the storage time was least in case of Peshawar while longest in case of Gilgit. The longer storage duration in Gilgit could be due to closure of roads in winter in the region.

**Table 7:** On-farm storage time (days) of different feedstuffs in different cities.

City	Cotton cake	Brassica cakes	Misc. Cakes^1^	Pulses^2^	Waste pulses^3^	Conc.	Grains^4^	Waste bread	Wheat bran	Pulse bran	Wheat Straw	Stover
Islamabad	17.8^c^	20.4^b^	—	—	—	23.0^a^	21.9^b^	18.5^a^	18.9^b^	17.5	25.9^cd^	20.0
Lahore	4.6^d^	1.0^b^	—	—	—	13.0^bc^	17.9^ab^	1.3^c^	5.0^e^	—	54.9^b^	1.0
Muzaff.	9.1^cd^	—	—	20.0	—	8.8^c^	23.7^ab^	7.0^bc^	8.3^de^	—	44.1^bc^	10.0
Karachi	15.7^bcd^	14.3^b^	11.2^b^	9.2	8.9^b^	7.6^c^	10.7^b^	5.6^c^	10.1^d^	9.7	12.1^d^	—
Peshawar	4.6^d^	6.7^b^	6.9^b^	—	—	7.7^c^	28.2^ab^	2.6^c^	5.5^e^	—	15.4^d^	—
Quetta	33.2^ab^	20.4^b^	53.3^a^	36.1	45.0^a^	14.4^ac^	19.2^b^	10.9^b^	14.6^c^	—	26.5^c^	—
Gilgit	—	102.9^a^	—	—	—	16.8^ab^	54.2^a^	4.7^c^	23.5^a^	—	115.8^a^	70.0
Total:	13.82	30.45	11.98	26.69	14.72	10.95	23.99	9.29	11.40	15.69	33.78	42.44
SD	33.60	60.93	15.37	28.83	17.48	9.88	49.93	10.21	11.14	8.60	54.13	55.44
*p*	0.001	0.019	0.000	0.240	0.000	0.000	0.044	0.000	0.000	0.176	0.000	0.492
*n*	245	40	63	16	25	100	124	167	305	13	324	9

*Means with different superscripts “^abcde^” differ significantly within a column at p < 0.05. Muzaff. = Muzaffarabad; Brassica cakes = mustard oilseed cake, taramira (Eruca sativa) seed cake, or canola meal; Conc. = Mixed commercial concentrates*.

1 *Includes maize, palm, and coconut oilcakes*;

2 *Includes Mung beans, cowpeas, or other single pulses*;

3 *Includes maize ad wheat grains*.

Despite the differences in on-farm storage time, association between the length of storage and aflatoxin levels in feedstuffs could not be established. The only case of positive association was of pulse bran, for which a longer on-farm storage in Islamabad compared to Karachi was associated with higher aflatoxin levels. The number of observations in this feedstuff were not enough to explore within city variations.

Longer storage under conducive conditions is positively correlated with aflatoxin development in foodstuffs (Achaglinkame et al., 2017). In this regard, relative humidity (Giorni et al., 2012; Pratiwi et al., 2015) and temperature (Schindler et al., 1967) during storage are important factors in affecting aflatoxin development. Maximum aflatoxin production occurs at 28–30°C (OBrian et al., 2007), and it ceases when temperature drops below 18°C (Schindler et al., 1967). In the present study, the lack of positive associations between storage length and aflatoxin contamination levels in feedstuffs could be because we collected samples in winter months. In particular, the cold weather of the Himalayan region (Gilgit and Hunza valley) explains the lack of effects of even more than 100 days of storage. In case of Quetta, where some feedstuffs were stored for over 1 month, the dry weather of the city seems to play a preventive role in aflatoxin development. The present results indicate that the storage time and conditions in the commercial feed stores might be more relevant in studying aflatoxin development in feedstuffs and should be included in future studies.

### Share of Feedstuffs in Aflatoxin M_1_

**Figure 2:**
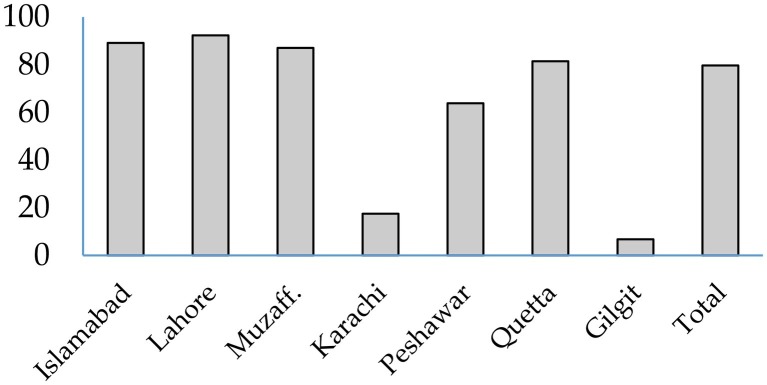
Share of aflatoxins from cottonseed cake in daily exposure to total aflatoxins in dairy animals in different cities.

Share of different feedstuffs in total daily aflatoxin exposure of animals in different cities were calculated. Overall, 79.7% of the aflatoxins consumed by dairy animals was coming from cottonseed cake. Cottonseed cake contributed 81–92% of daily aflatoxin exposure of animals in the peri-urban farms located in Islamabad, Lahore, Muzaffarabad, and Quetta (Figure 2). The share of cottonseed cake in cities of Karachi and Peshawar was 20 and 64%, respectively. In case of Peshawar, 21% of total daily aflatoxin consumed by animals was contributed by bakery waste. Cottonseed cake waste and bakery waste were together responsible for 90.2% of the daily aflatoxin exposure of animals in Peshawar. The aflatoxin exposure of dairy animals in the city of Karachi was from many feed ingredients including grains (19%), waste pulses (13%), and waste bread (8%).

## Conclusions

It may be concluded from the present results that the AFM_1_ levels in milk produced in peri-urban dairy farms, except in Gilgit, are exceptionally high. Around 80% of the AFM_1_ in milk was found to be contributed by cottonseed cake in dairy rations. The high milk contamination can therefore be reduced by replacing cottonseed cake with feedstuffs lower in aflatoxin contamination such as canola meal, and commercial concentrate feeds. Long-term mitigation strategies should focus on reducing aflatoxin contamination in cottonseed cake and discouraging use of bakery waste as dairy animal feed.

## Data Availability Statement

The datasets generated for this study are available on request to the corresponding author.

## Author Contributions

AY and MI contributed to conceptualization, visualization, funding acquisition, and supervision. AY, AmU, and JL contributed to methodology. AY contributed to questionnaire formulation, data curation, project administration, and original draft preparation. AY, MI, AmU, ZA, AtU, SS, AZ, HI, SJ, ZF, AH, BA, ZB, and IK contributed to sample collection. AY, NI, and ZA contributed to method validation. AY, ZA, AtU, MA, UF, and AA contributed to laboratory analysis. AY, AmU, and JL contributed to writing-review and editing.

### Conflict of Interest

The authors declare that the research was conducted in the absence of any commercial or financial relationships that could be construed as a potential conflict of interest.
